# Bacterial Cellulose Properties Fulfilling Requirements for a Biomaterial of Choice in Reconstructive Surgery and Wound Healing

**DOI:** 10.3389/fbioe.2021.805053

**Published:** 2022-02-11

**Authors:** Jerzy Jankau, Agata Błażyńska‐Spychalska, Katarzyna Kubiak, Marzena Jędrzejczak-Krzepkowska, Teresa Pankiewicz, Karolina Ludwicka, Aleksandra Dettlaff, Rafał Pęksa

**Affiliations:** ^1^ Department of Plastic Surgery Medical University of Gdańsk, Gdańsk, Poland; ^2^ Institute of Molecular and Industrial Biotechnology Lodz, University of Technology, Łódź, Poland; ^3^ Bowil Ltd, Władysławowo, Poland; ^4^ Department of Pathology, Medical University of Gdansk, Gdansk, Poland

**Keywords:** bacterial cellulose (BC), reconstructive surgery, wound dressing, mechanical properties, biocompatibility and biodegradability

## Abstract

Although new therapeutic approaches for surgery and wound healing have recently made a great progress, there is still need for application of better and use novel methods to enhance biocompatibility as well as recovery and healing process. Bacterial Cellulose (BC) is natural cellulose in the form of nanostructure which has the advantages of being used in human body. The medical application of BC in reconstructive, cardiac and vascular surgery as well as wound healing is still under development, but without proved success of repetitive results. A review of studies on Bacterial Cellulose (BC) since 2016 was performed, taking into account the latest reports on the clinical use of BC. In addition, data on the physicochemical properties of BC were used. In all the works, satisfactory results of using Bacterial Cellulose were obtained. In all presented studies various BC implants demonstrated their best performance. Additionally, the works show that BC has the capacity to reach physiological as well as mechanical properties of relevance for various tissue replacement and can be produced in surgeons as well as patient specific expectations such as ear frames, vascular tubes or heart valves as well as wound healing dressings. Results of those experiments conform to those of previous reports utilizing ADM (acellular dermal matrix) and demonstrate that the use of BC has no adverse effects such as ulceration or extrusion and possesses expected properties. Based on preliminary animal as well as the few clinical data BC fittings are promising implants for various reconstructive applications since they are biocompatible with properties allowing blood flow, attach easily to wound bed and remain in place until donor site is healed properly. Additionally, this review shows that BC can be fabricated into patient specific shapes and size, with capability to reach mechanical properties of relevance for heart valve, ear, and muscle replacement. Bacterial cellulose appears, as shown in the above review, to be one of the materials that allow extensive application in the reconstruction after soft tissue defects. Review was created to show the needs of surgeons and the possibilities of using BC through the eyes and knowledge of biotechnologists.

## 1 Introduction

For the last years, the main method of treating diseased or dying soft tissues was to remove them, which significantly compromised the patient’s quality of life. Reconstructive options from the patient’s own tissues are limited, and no materials were widely available to replace the removed tissues, resulting in little opportunity for reconstruction. This situation has improved significantly in recent decades, largely due to the progress of biotechnology and the possibility of using better and compatible materials to replace diseased tissue. The use of biomaterials is nowadays increasingly common both *in vitro* and *in vivo*, but there are still many areas of medicine where further research using biomaterials is needed.

Biotechnological developments are increasingly more often used in surgery. This is mainly because the patient’s own tissues do not always allow full functionality and thus do not fulfil that task. An alternative is to obtain the material from a living donor, but this means that patients need to be started on regimens reducing the risk of tissue rejection. Their use is also associated with very long and complicated procedures and thus with very high costs. That is why new materials to replace living tissues are so much sought after. Their use is possible thanks to such properties as bioinertness, adequate mechanical strength, ease of shaping and modification, nanoporosity or hydrophilicity (Pang et al., 2020). Especially natural biopolymers have gained acceptance in medical practice. They show low or no immunogenicity (particularly when based on polysaccharides), and they are favorably perceived by the public, which is also significant. Many *in vitro* laboratory tests, *in vivo* tests and clinical trials, have indicated that biomaterials can be an adequate mechanical and functional replacement for the living structures of the patient’s tissues. New bioimplants are increasingly widely used to fill in defects and foster healing and regeneration of the surrounding tissues.

This study focuses on the specific features of one biomaterial, bacterial cellulose (BC). Contrary to its plant equivalent, it is formed as a pure polymer hydrogel without lignin admixtures, which must be removed from the plant cellulose mass. Bacterial cellulose nanofibers are characterized by a high concentration of hydroxyl groups and a surface with densely packed thin fibers. Unlike plant-derived cellulose, whose fibers are much thicker and have a lower hydrogen bond density, BC is more hydrophilic and has a more remarkable ability to form a three-dimensional branched network of ultra-thin fibers. This structure determines many beneficial properties, such as high tensile strength, excellent water holding capacity (98%), which, in turn, spark an interest in bacterial cellulose as a biomedical material of natural origin (Pogorelova et al., 2020).

### 1.1 The Natural Diversity of BC and Its Potential Applications in Medicine

Bacterial cellulose is atypical biomaterial used in medicine due to its biotechnological origin, most of its features may be controlled by appropriate choice of a producer strain and conditions for propagation. The great majority of examples of BC applications in medicine include the polymer produced by *Komagataeibacter* as well as *Zoogloea* genus of bacteria. Cellulose synthesizing bacteria colonize rotting fruits and other parts of plants, hence their presence in the production processes of traditional fermented products (such as Nata de Coco dessert or Kombucha, Kimchi drinks) (Nugroho and Aji, 2015; Villarreal-Soto et al., 2018) in the form known as SCOBY (symbiotic culture of bacteria and yeast). The variety of environments from which BC-producing strains are isolated generates a variety of strains with different living requirements. For example, glucose is the preferred carbon source for most strains, e.g., for *K. xylinus* E26, *K. hansenii* 53582, *K. hansenii* 23770 (Ryngajłło et al., 2019b; Chen et al., 2019), but there are examples of strains that also efficiently grow on sucrose, for instance, *K. hansenii* 23770 (Chen et al., 2019) or glycerol, such as, e.g., *K. xylinus* E26 (Ryngajłło et al., 2019b). The presence of ethanol in the culture medium is most often desirable, as is the case for the strains *K. xylinus* E25 and, e.g., *K. xylinus* subsp. *sucrofermentans* BPR3001A, *K. rheticus* K3 or *K. uvaceti* FXV3, for which the addition of 2% v/v ethanol to the medium causes up to a twofold increase in the cellulose synthesis efficiency (Naritomi et al., 1998; Krystynowicz et al., 2002; Jacek et al., 2021; Nascimento et al., 2021) but in some cases, it reduces cellulose production, e.g., in the case of the strain *K. hansenii* 53582 (Jacek et al., 2021). The differences in breeding preferences of the *Komagataeibacter* strains and their sensitivity to environmental factors result from the genetic diversity of cellulose producing bacteria and are reflected in the properties of the biopolymer they produce (Ryngajłło et al., 2019a; Ryngajłło et al., 2019b). The latter aspect is vital in selecting the appropriate producer strain for the intended use of the material. Resent studies has proven that carbon source used for cellulose propagation has an influence on membrane morphology, namely medium with the highest sucrose content resulted in evident pore size reduction (from 17.832 ± 4.429 µm to 2.698 ± 1.035 µm) and tensile strength increase (from 240.75 ± 14.62 MPa to 339.04 ± 33.82 MPa) in the BC produced by *K. xylinus* strain TISTR 975 (Jaroennonthasit et al., 2021).

Depending on the strain of the genus *Komagataeibacter* used in cultivation, the obtained cellulose membranes differ in flexibility, water holding capacity (WHC), cellulose content, degree of polymerization and degree of crystallinity (Table 1). The texture of BC hydrogels is determined both by the network of cellulose fibers and by the water contained in the membrane. It can be observed that the higher the cellulose content or density, the lower the WHC value, and the higher the values of BC breaking stress (Table 1). Cellulose is biosynthesized by cellulose synthase enzyme complexes (terminal complexes, TCs) arranged along the long axis of the cell (Brown et al., 1976; Zaar, 1979; Benziman et al., 1980). Cellulose fibers that build membranes are ribbons coiled from nanofibers drawn from all TCS of a given cell (Benziman et al., 1980). Therefore, most likely, the producer cell morphology translates directly into such properties of cellulose fibers as their diameter or nanofiber packing density. The tendency (or lack thereof) of bacterial cells to move may affect the spatial arrangement of the fibers. In turn, their morphology (diameter, shape and spatial arrangement) affects the mechanical strength of the synthesized material. Machado et al. (2016) observed that the BC synthesized by *K. xylinus* ATCC 23760 is made of fibers with an average size of about 150 ± 50 nm, while the BC membrane synthesized by *K. rhaeticus* is made of fibers approx. 100 ± 25 nm in size on average (Machado et al., 2016). Young’s tensile modulus for *K. rhaeticus* BC was twice as high as that of *K. xylinus* ATCC 23760 BC (Table 1). As can be seen from the above examples, even slight differences between the cells of producer strains translate into the properties of the material they produce, such as the width and packing density of cellulose fibers (and thus the pore size), which in turn translates into the absorption capacity (water holding capacity, WHC), water retention capacity (WRC) and the mechanical properties of the formed membrane (Chen et al., 2017; Vigentini et al., 2019). Therefore, the knowledge about the diversity of bacterial strains which efficiently synthesize cellulose differing parameters as well as the multiplicity of changes and additional modifications that can be designed for this biopolymer as early as at the stage of bacterial cultivation and post-culture processing allow for extending the application possibilities of this material is evidenced by the emerging new research results in the field of *in vivo* tests and clinical trials of dressings and implants based on bacterial cellulose.

**TABLE 1:** Characteristics of BC produced by different strains of *Komagataeibacter* genus.

Bacterial strain	Cellulose concentration (wt%)	Density (g/cm^3^)	Water holding capacity (WHC)	Crystallinity (%) ± 5	Iα fraction (%) ± 2	Breaking stress (MPa)	Apparent Young’s modulus (MPa)	References
*K. xylinus* ATCC 700178	.19 ± .1	.0024 ± .001	(5.26 ± .35) × 10^4^ (%)	83	66	.15 ± .08	1.10 ± .38	(Chen et al., 2017, 2018)
*K. xylinus* ATCC 10245	.18 ± .1	.0024 ± .001	(5.44 ± .27) × 10^4^ (%)	86	56	.36 ± .08	2.87 ± 1.33	
*K. hansenii* ATCC 23769	.22 ± .1	.0031 ± .001	(4.50 ± .20) × 10^4^ (%)	79	48	.12 ± .04	1.26 ± .66	
*K. xylinus* NBRC 13693	.6 ± .1	.0069 ± .001	(1.65 ± .15) × 10^4^ (%)	83	57	.62 ± .17	3.08 ± .66	
*K. xylinus* ATCC 53524	.72 ± .1	.01 ± .002	(1.37 ± .11) × 10^4^ (%)	80	60	.68 ± .13	5.56 ± 2.29	
*K. xylinus* KTH 5655	.42 ± .1	.0045 ± .001	(2.35 ± .18) × 10^4^ (%)	84	66	.62 ± .16	3.83 ± 1.08	
*K. hansenii* LMG 1527	nd.	nd.	25.9 ± 5.8 (g_water_/g_cellulose_)	75	84	nd.	nd.	Vigentini et al. (2019)
*K. nataicola* LMG 1536	nd.	nd.	22.5 ± 9.1 (g_water_/g_cellulose_)	80	89	nd.	nd.	
*K. rhaeticus* LMG 22126	nd.	nd.	18.7 ± 4.5 (g_water_/g_cellulose_)	72	84	nd.	nd.	
*K. swingsii* LMG 22125 isolate GSG	nd.	nd.	42.3 ± 2.2 (g_water_/g_cellulose_)	65	93	nd.	nd.	
*K. swingsii* LMG 22125 isolate GSP	nd.	nd.	34.5 ± 5.9 (g_water_/g_cellulose_)	64	67	nd.	nd.	
*K. xylinus LMG* 1515	nd.	nd.	10.7 ± 1.1 (g_water_/g_cellulose_)	77	86	nd.	nd.	
*K. xylinus LMG* 1518	nd.	nd.	20.7 ± 3.5 (g_water_/g_cellulose_)	74	84	nd.	nd.	
*K. xylinus ATCC 23760*	nd.	nd.	∼128 (g_water_/g_cellulose_)	85	nd.	41.5 ± 4	1.5 ± .2 ×10^3^	(Machado et al., 2016)
*K. rhaeticus*	nd.	nd.	∼68 (g_water_/g_cellulose_)	83	nd.	46.9 ± 3	3.2 ± .3 ×10^3^	

Native cellulose was first used as a moist dressing for burn and ulcerative wounds (Czaja et al., 2006). However, it soon transpired that its unique compatibility with human tissues opened many more possibilities for its use in biomedicine. As a non-degradable implant, indifferent to the surrounding tissues, cellulose has been used to replace the dura mater, eardrum, valve or interventricular septum. Cellulose biomaterials provide adequate tissue protection in these areas, primarily due to stability, high mechanical strength and flexibility, high hydrophilicity and excellent oxygen permeability (Rosen et al., 2011; Lang et al., 2015; Czaja, 2020; Dawidowska et al., 2020).

Bacterial cellulose reinforced *in situ* (during cultivation) with a polymer or titanium mesh can be used where higher mechanical strength of the biomaterial is required, in herniotherapy or bone reconstruction procedures (Ludwicka et al., 2018). The structure is also strengthened by the specific arrangement of the fibers (Wang et al., 2017). Cellulose produced by techniques involving a wet-drawing and wet-twisting process of ultralong nanofibers shows significantly greater stiffness, as compared with natural cellulose fibers, regenerated cellulose macrofibers, and novel lightweight steel materials, as well as reduced porosity, both resulting from a highly ordered orientation of fibers.

By controlling cellulose shape during cultivation or adjusting it after cleaning, we extend the scope of its applications to vascular surgery and neurosurgery, including, in particular, tubes for vascular reconstruction or peripheral nerves regeneration (neurotubes) (Kowalska-Ludwicka et al., 2013; Bao et al., 2020). The appropriate nanostructure of the material means an appropriate range of limited (selective) permeability and flexibility adjusted to the requirements applicable to vascular implants and guidance channels (Kołaczkowska et al., 2019).

Compacted (densified) cellulose BC (about 17%) is a competitive scaffolding material for the reconstruction or regeneration of ear cartilage or bone tissue. This kind of material corresponds to the elastic mechanical properties of human cartilage and can be produced in patient-specific auricular shapes, and at the same time shows excellent biocompatibility with chondrocytes and osteocytes *in vivo*, without interfering with the healing processes (Nimeskern et al., 2013; Martínez Ávila et al., 2015; Pang et al., 2020). Several techniques have been developed to aid cell growth into BC scaffolds, such as adjusting the pore size and pore interconnection during BC (Ashrafi et al., 2019; Zhang et al., 2019), and at the post-production stage—through laser ablation (laser patterning) (Jing et al., 2013), 3D printing (Sultan et al., 2017), or freeze-drying (Martínez Ávila et al., 2015). Particular attention is paid to the methods that increase the microporosity of cellulose without adversely affecting its mechanical and biological parameters, as they frequently constitute the exceptional value of this biopolymer.

The mentioned nanoporosity, hydrogelicity, large surface area and excellent loading capacity of cellulose are successfully used in drug delivery systems (DDS). Such carriers can be used in many fields of pharmacy, medicine and surgery, from dressings to internal or dental implants that deliver painkillers and anti-inflammatory drugs or bacteriostatics to a specific location (Badshah et al., 2017).

The aim of this review is to draw attention to the growing number of applications of bacterial cellulose in regenerative medicine, which have been validated in clinical trials or *in vivo* experiments. This article also discusses the specific properties of BC to pave a way to increasing its recognition on the market of implants and dressings. The presented examples of BC applications relate only to the most advanced and practically relevant research. The paper indicates the ways of selecting or modifying the properties of BC in terms of specific requirements set by specialists in reconstructive medicine. We have limited the scope of this paper to two areas: reconstructive surgery and wound treatment as the fields with the best-documented applications of BC.

## 2 Reconstructive Surgery

### 2.1 Requirements for Implants

Reconstructive surgery is a surgical field whose representatives face the challenge of closing various defects in many body regions. They include extensive, post-traumatic defects of muscles or soft tissues covering them, and minor ones, where they constitute a frame or support. Thanks to the intensive development of technology involving the production of new biomaterials, reconstructive surgery has changed a lot in recent years. New implants are more widely used to fill in defects and foster healing and cell production in the surrounding tissues.

Tissue replacement materials should have the following basic properties:

1. no signs of antigenicity,
2. lack of toxicity,
3. preventing contracture or scarring and provoking the lowest possible inflammatory response,
4. sterility and bacterial barrier or antibacterial properties,
5. appropriate water volume and appropriate mechanical properties, depending on the type of reconstructed tissue,
6. facilitating angiogenesis.

In addition, it is good if they are readily available, packaged and handled, and the production and storage costs are low. It is not easy to implement because many of these features can be mutually exclusive, e.g., mechanical strength with adequate flexibility and maximum reduction of the material thickness. The set of desired features depends on the field of surgery and the properties of the tissues at the site of intervention. The reconstruction of cartilage requires a different set of properties than bone reconstruction. However, BC offers vast possibilities and developing one advantage can be prioritized over another. A different aspect that is very important in cardiac surgery or vascular surgery is the production of long-lasting materials that do not increase the tendency to platelet aggregation and, as a result, thromboembolic complications and are resistant to long-term repetitive work.

### 2.2 BC as an Implant

A field where biomaterials are already used successfully is reconstructive surgery of the integumentary structures. The potential for a controlled change in the properties of bacterial cellulose depending on the method and conditions of cultivation or post-biosynthetic modification makes it especially suitable (Kołaczkowska et al., 2019) for soft tissue reconstruction. The requirements for the expected properties of each reconstructed tissue are vast and diverse. This is mainly due to the physiological functions of given tissues. The obtained BC material may have properties suitable for an implant of elastic skin and a cartilage scaffold, for instance. There are increasingly more described applications in many areas of reconstructive surgery (Table 2).

**TABLE 2:** Examples of applications of BC implants in reconstructive surgery.

Reconstructive surgery field	Application description	BC type and modification	Stage of research	Benefits of using BC	Literature
Cardiovascular surgery	BC conduit as small-caliber vascular prosthesis	• wet and dry tubes • smoothness of inner surface improvement • application of antiplatelet therapy	Animals • rabbits, carotid artery • sheep, carotid artery	• excellent anticoagulant properties and cells compatibility	(Weber et al., 2018; Wacker et al., 2020; Bao et al., 2021; Klemm et al., 2021)
				• good tensile strength and suture retention	
				• reduced thrombogenic	
				potential for smoother BC surface	
				• improved patency rates for BC tubes receiving antiplatelet therapy	
				• modification of BC with hyaluronic acid triggered the inflammatory reaction	
	BC patch for blood vessel reconstruction	• BC patch • BC composite with hyaluronic acid	Animals • pigs, walls of jugular vein and jugular artery		(Kołaczkowska et al., 2019; Osorio et al., 2019; Klemm et al., 2021)
Neurosurgery	BC nerve conduit (tube), regeneration of peripheral nerves	• wet tubes • native and mercerized tubes	Animals • rats, facial nerve and femoral nerve	• BC easily shaped into a hollow tube guided nerve axons, resulting in better nerve regeneration after transection	(Kowalska-Ludwicka et al., 2013; Binnetoglu et al., 2020)
				• reduction of inflammatory reaction and neuroma formation	
	BC membrane as dura mater substitute	• native BC • electrospun BC	Animals • rats, rabbits; dural defects	• retaining the properties of local tissues and providing adequate mechanical properties, without the need for sutures	(Lima et al., 2017a; Jing et al., 2020)
				• no induced immune reaction, nor chronic inflammatory response, absence of neurotoxicity signals	
				• electrospun BC implantation showed more collagen fibers evenly distributed on the outer side of implants, fewer brain tissue adhesions and epidural scars were found	
	BC-based intervertebral disc	• 3D micropatterned BC	Animals • rats, total disk implantation in caudal spine 3/4	• excellent structural (shape maintenance, hydration, tissue integration) and functional (mechanical support and flexibility) performance	Yang et al. (2018)
				• controlled cellular alignment	
General surgery	BC based surgical mesh, abdominal muscle aponeurotic defect, hernia repair, soft tissues reinforcement, antiadhesive material	• BC composite with chitosan and/or polypropylene mesh • compact and perforated BC membrane • single-layer dry BNC patches, multi-layered BNC meshes in combination of BNC with standard polypropylene meshes	Animals • rats: implantation into the pocket of panniculus carnosus muscles along the dorsal midline; muscle aponeurotic defect reconstruction; intraperitoneal implantations • rabbits, implantations onto the abdominal wall between the peritoneum and the visceral organs	• reduced immune response	(Coelho Junior et al., 2015; Silveira et al., 2016b; Piasecka-Zelga et al., 2018; Anton-Sales et al., 2021)
				• induced tissue remodelling	
				• no connective tissue proliferation in nearby muscle structures	
				• superior to common polypropylene and ePTFE meshes biological integration with surrounding tissues	
				• reduced peritoneal adhesions as compared to standard synthetic meshes	
				• BC laminates with 2 or 3 films were resistant enough to reach the minimal acceptance criteria for abdominal wall reinforcement applications	
				• BC exhibited favourable surgical features in terms of saturability, manageability and accommodation to the implantation site	
	BC mesh for pelvic floor reconstruction following implantation in the vagina	• native BC membrane	Animals • sheep, implantation in the submucosa of the posterior vagina wall	• biomechanical characteristics and tissue remodelling of the BC mesh met the basic requirements of pelvic floor reconstruction	Ai et al. (2020)
				• negative effect: BC induced greater inflammatory response than standard Gynemesh™ implant	
	BC membrane as a protective barrier to prevent urethral damage after implantation of artificial devices	• native BC membrane	Animals • rats, strip of the BC applied around the urethra below the bladder neck	• integration with the surrounding tissue, contributing to its architecture remodelling and strengthening	Lima et al. (2017b)
				• the obtained level of collagen deposition parameters, vascularization and structural increase in urethral wall thickness may represent new perspective for longer survival of artificial implants	
	BC membrane to reinforce urethrovesical anastomosis	• perforated native BC membrane	Animals • rabbits, urethrovesical anastomosis with BC reinforcement	• absence of extrusion, stenosis or urinary fistula	Maia et al. (2018)
				• good biocompatibility and biointegration with tendency to the urothelial wall thickening	
	BC gel to revert the loss of anal resting pressure after anorectum sphincter injury (fecal incontinence)	• hydrated BC gel	Animals • rats, sphincter injury followed by BC gel injection	• BC presented the ideal characteristics as bulking agent	Cavalcante et al. (2018)
				• increased anorectal resting pressures were observed	
				• BC promoted neovascularization, the implant area was colonized by multinucleated giant cells, fibroblasts and dense conjunctive tissue associated to collagen fibres	
	BC film for reparation of bile duct injury	• native BC film	Animals • pigs, reconstruction of common bile duct defect	• BC proved to be a biocompatible material that produced a complete healing process and biliary flow continuity	de Abreu et al. (2020)
				• a compact nonporous BC structure prevented leakage of bile	
Laryngology	BC graft in closure of tympanic membrane perforation (myringoplasty)	• dried BC membrane	Human • BC put over the perforation and lateral to the tympanic membrane remnant	• 100% healing in patients with BC graft	(Silveira et al., 2016a; Mandour et al., 2019)
				• BC graft myringoplasty was a good, simple, rapid and safe surgery that could be done under local anesthesia in outpatient clinic with shorter time of surgery than fat graft myringoplasty and temporalis fascia graft myringoplasty, with better hearing and healing	
				• BC graft functions as an inducer of tissue remodelling and as a promoter of the healing process, by enabling an intensive process of revascularization and epithelialization, which might explain the regeneration of the eardrum remains and also the closure of tympanic membrane	
	BC membrane for pharyngocutaneous fistula closure after laryngectomy	• native BC membrane	Animals • rats, pharyngoesophagotomy closed with BC or sutures and BC	• fistula closure was significantly better in BC with primary sutures group	Demir et al. (2018)
				• BC promoted fibroblasts proliferation, which was significantly higher in the group treated with both BC and primary sutures	
	BC graft material in correcting and preventing dorsal nasal disorder in rhinoplasty	• native BC membrane	Animals • rats, shredded cartilage wrapped in BC and placed in a subcutaneous area at the back	• good cartilage health and integrity	Aydinli et al. (2017)
				• negative effect: significantly lower degree of vascularization and fibrosis and greater degree of chronic inflammation for BC implants	
Other	BC membrane for trapping tumor cells in glioblastoma treatment, implantation after surgical resection	• native BC discs	Animals • rats, implantation in brain parenchyma	• BC was a biocompatible scaffold that could trap tumor cells	Autier et al. (2019)
				• high flexibility made it easy to introduce into the tumor bed after resection and its visibility on MRI may facilitate stereotactic radiosurgery	
				• BC could be easily loaded with chemoattractants	

### 2.3 Examples of BC Implant Application in Clinical Practice and *In Vivo* Experiments

**FIGURE 1:**
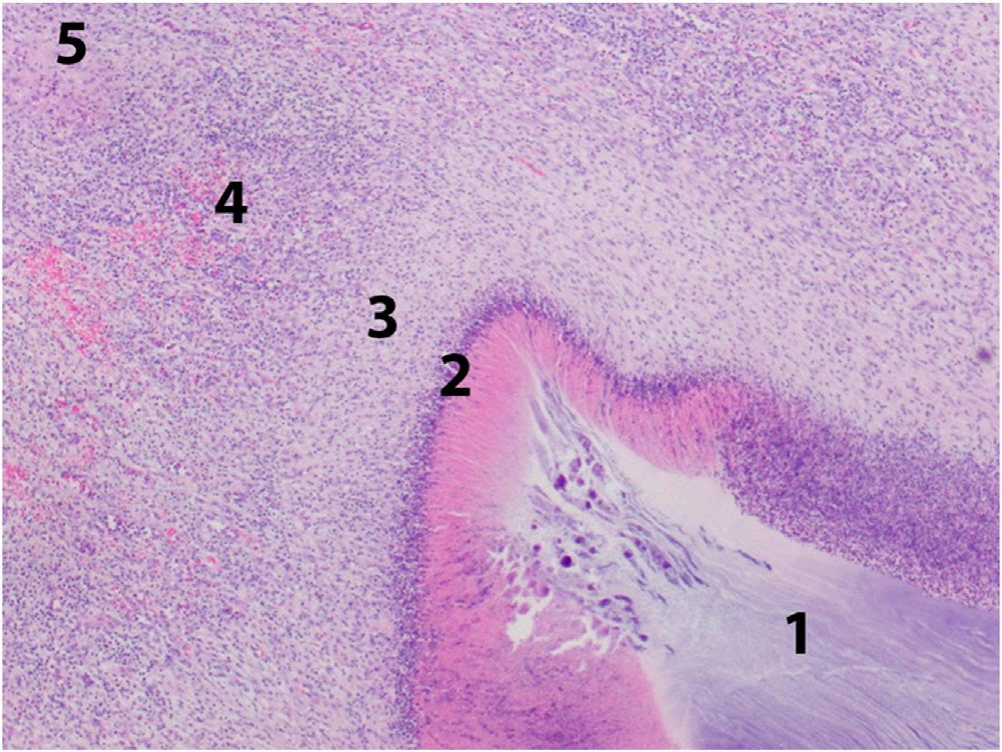
Body integument - resorptive reaction to an implant. Zone 1: implant, zone 2: purulent necrosis, zone 3: macrophagal infiltration, zone 4: granulation tissue, zone 5: fibrous scar tissue.

**FIGURE 2:**
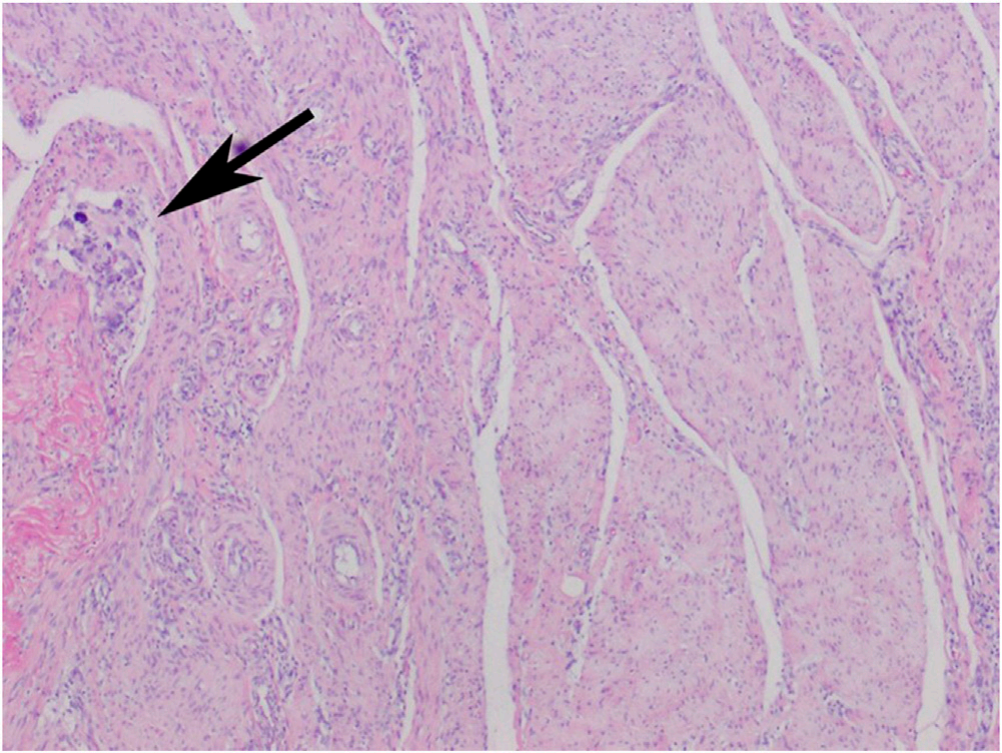
Body integument. Fibrous scar tissue formed by thick collagen-fibroblastic bundles. Focal resorptive granuloma (arrow).

**FIGURE 3:**
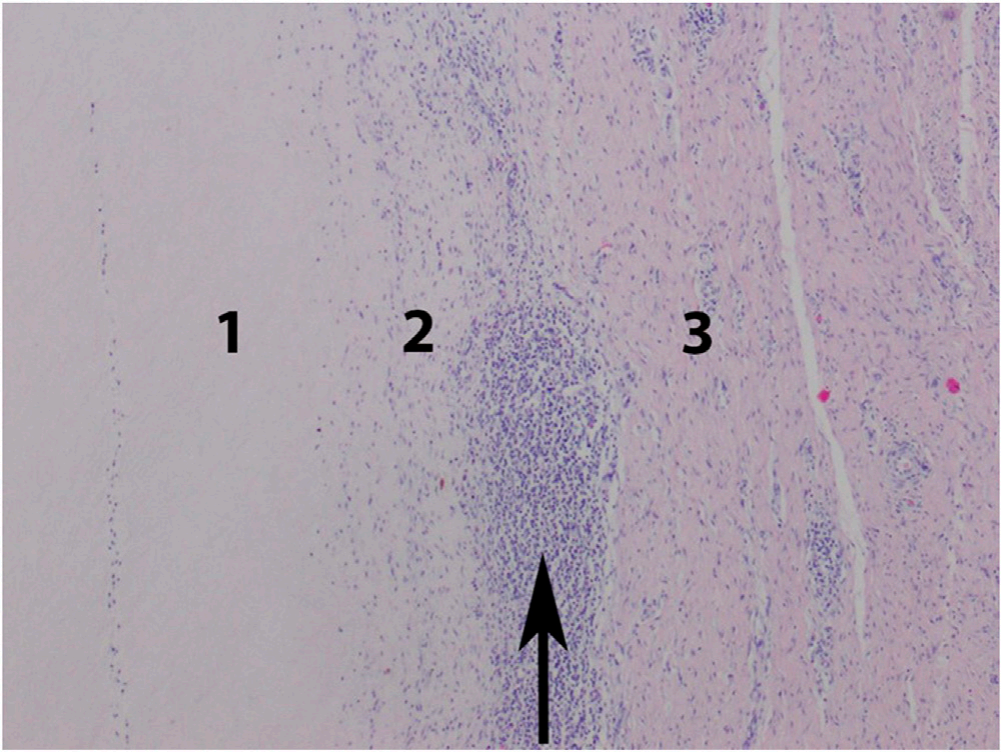
Auricle. Resorptive reaction to the implant. Zone 1: implant, zone 2: macrophagal infiltration, zone 3: fibrous scar tissue. Focal nodular cluster of lymphocytes (arrow).

**FIGURE 4:**
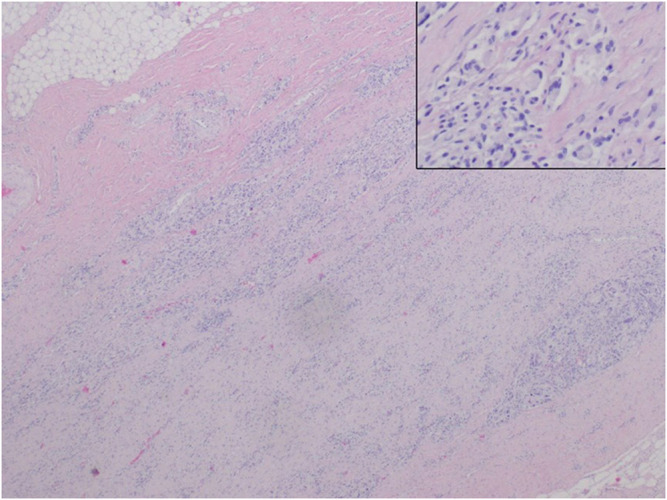
Auricle. Interstitial granules (insert) are visible between the thick bundles of fibrous scar tissue.

BC has been widely described in many surgical fields. Bacterial nanocellulose patches are excellent biological dressings serving as a temporary skin substitute when applied to exudative and bleeding tissues (Kwak et al., 2015). This is important in wounds with a significant loss of tissue, such as burns or ulcers. Moreover, BC is an excellent scaffold for type I collagen synthesis by mesenchymal stem cells (Vielreicher et al., 2018). A detailed analysis with collagen I-specific binding protein revealed a highly ordered collagen network structure at the interface between the cell and bacterial nanocellulose. This shows the reproductive capacity of soft tissues thanks to BC. The potential of bacterial nanocellulose in integumentary reconstruction was also assessed *in vivo* (Jankau et al., Unpublished study). The study was conducted on three pigs. The same defects were produced in the ear cartilage and the rectus abdominis muscle, and BC membranes were sutured into them. After 3 months, the morphology, physical parameters and functions of BC were assessed compared to the live body tissues. Histopathological examinations included the degree of resorption, pus exudation, fibrosis and scar formation (Figures 1–4). The implantation sites did not show clinical signs of complications such as inflammation or necrosis. Histologically, a normal scar was produced due to the material healing into the host’s body. These preliminary studies confirmed the usefulness of BC as an effective filling of cartilage and muscle tissue defects.

BC can be used as a cartilage implant. Until now, the gold standard of ear cartilage reconstruction was the use of autologous rib grafts, which significantly extended the operation time, increased the surface area of the patient’s injured tissue and was associated with a higher risk of complications. The development of tissue engineering is a promising alternative therapy for this approach. BC has also been reported as a material for the reconstruction of vestibular cartilage. Excellent biocompatibility and tissue integration were demonstrated (Martínez Ávila et al., 2015). BC scaffolds provide good mechanical stability and maintain structural integrity while providing a porous architecture that supports chondrocyte development.

Another area where bacterial nanocellulose is used as a reconstructive material is ophthalmology. Corneal infection or improper treatment can have serious consequences, such as vision deterioration, vision loss, and deteriorated quality of life. A method for treating such injuries has been developed. It involves using biomaterials as a dressing for the damaged cornea (Anton-Sales et al., 2021). The use of an amniotic membrane graft was compared to the use of bacterial nanocellulose. BC was shown to be effective in covering corneal defects. Moreover, unlike the amniotic membrane, BC is stable at room temperature, and its production is much easier—the size, shape and thickness are much easier to control. In addition, BC can be easily sterilized by heat. Its use is tissue-independent and carries no risk of disease transmission as it is a product of plant origin.

Bacterial cellulose can also be used in neurosurgery. A frequent complication of head injuries, neurosurgical procedures, congenital defects or neoplastic diseases is disruption or damage of the dura mater. The primary function of the dura mater is to protect the central nervous system and provide an appropriate environment; therefore, disrupting its integrity is a significant problem in neurosurgery. The material that can be replaced must be biocompatible and cannot cause inflammation because the potential activation of inflammatory mediators, including nitric oxide or prostaglandins, may lead to a cascade of inflammatory processes and, as a result, to inflammatory diseases of the central nervous system (CNS), which are associated with a high risk of long-term complications. Until now, silk fibroin, human amniotic membrane and other materials have been researched as substitutes for dura mater. The BC has the advantage of being cost-effective and straightforward to produce. Several papers describe the repair of dural damage with BC membranes (Goldschmidt et al., 2016). They indicate a great potential of BC for use as a new type of artificial dura mater material due to its high strength and very good biocompatibility. The commercial production of the material is already planned (Bacakova et al., 2019; Lipovka et al., 2021).

Congenital heart defects are among the most common defects and the most common cause of death in children under 1 year. Ventricular septal defect accounts for the highest percentage of congenital heart defects (20%–29%). It is usually closed with a synthetic patch (PTFE), an autologous or bovine pericardium patch, but long-term results do not give satisfactory results. Currently available materials are not sufficiently flexible, which may weaken the diastolic compliance of the heart in the long term. Therefore, a biomaterial is sought to combine biocompatibility, adequate flexibility, tensile strength and flexibility similar to heart tissue. The network of cellulose nanofibrils shows similarities to the collagen network, thanks to this biomaterial has high mechanical strength, favorable flexibility and.

Water content up to 99%. A study was conducted on 25 pigs (German landrace pigs) (Lang et al., 2015), in which the ventricular septal defect was closed with BC sheets. Immediate occlusion was observed in all cases, and no tendency for platelet aggregation was detected. The biocompatibility of BC was good in the presence of blood, and the tissue responses were comparable to the materials currently used. Wound healing was observed to be normal, with complete neoendotheliation, progression over time with cellular organization, and mild chronic inflammatory reactions. The use of BC in vascular surgery or cardiac surgery is currently being explored by several research centers worldwide. Chemically pure BC free of pyrogenic agents is produced by Bowil Biotech Sp. z o.o., Władysławowo, Poland, and used in animal models as part of the “Kardio-BC” study (Kołaczkowska et al., 2019). Large animals such as pigs are often used in preliminary biocompatibility studies of new materials developed for use in medicine, including vascular and cardiac surgery. They were used in studies involving the implantation of reconstructive pericardial patches, thoracic aortic prostheses or both to determine BC biocompatibility (Kowalik et al., 2020). Individual operations were performed sequentially, depending on the increasing surgical complexity: pericardial patches, thoracic aortic prostheses, and a combination of both. After 90 days of observation, the animals were euthanized, and tissue fragments containing BC implants were collected at necropsy and subjected to histopathological examination. The assessment of bioimplant properties included macro and microscopic evaluation, scanning electron microscopy (SEM), X-ray diffraction examination, histopathological evaluation of the inflammatory reaction of the surrounding tissues. These studies provided evidence for the usefulness of BC as a vascular prosthesis in an animal model. The results indicate adequate healing of the prostheses, low percentage of mechanical damage, no infiltration of the host cells inside bacterial cellulose bioimplant, coverage of the bioimplant surface by the native vascular endothelium. The material itself did not degenerate. In addition, it was more durable than natural tissues such as porcine pericardial sac, aortic valve cusps or aortic wall.

Similar in vascular surgery, a material imitated blood vessels (creates thin “tubes”) that will no affected blood clotting) has been searched for years. Just as the use of two dimensional (2D) biomaterials has already bee relatively thoroughly studied, three dimensional (3D) BC is still unknown topic. Some 3D BNC biomaterials have been reported in the literature, but rather there are theoretical pages about properties (Klemm et al., 2021). There is described the *in vivo* use of BC by Osorio et al., evaluating the effect of using BC as blood vessels and their haemolysis and thrombogenicity (contact coagulation) (Osorio et al., 2019). The presented research results, it can be concluded that bacterial cellulose can be safely and effectively used as a vascular prosthesis and heart valve prosthesis after appropriate preparation.

## 3. BC Application in Healing Wounds

The healing process of all kinds of injuries and wounds, both internal and external, is the result of many coordinated molecular mechanisms, and their correct regulation is crucial for the duration and results of treatment. The risk of bacterial or fungal infection and an incorrectly progressing inflammatory process may also affect the outcome. If the healing process is normal, haemostasis (bleeding) is followed by an inflammatory reaction lasting from 24 h up to 6 days. The immune system cells must release cytokines and proteolytic enzymes to cleanse the wound of damaged tissue debris and prevent secondary infection by producing reactive oxygen species (ROS) (Thomason et al., 2018). Topical application of antibiotics and anti-fungal substances are therefore essential for treatment success. In addition, natural signaling molecules involved in healing processes, such as cytokines, components of the extracellular matrix (ECM) or tissue-specific growth factors, are now used as active substances in modern dressing and regeneration materials (Catanzano et al., 2021; Farahani and Shafiee, 2021). What is particularly challenging in dressing materials is the treatment of chronic and hard-to-heal wounds in which the balance between matrix metalloproteinases (MMPs) necessary for cleaning damaged tissue and their inhibitors (tissue inhibitors of metalloproteinitors, TIMPs) is disturbed (Grzela et al., 2016; Dissemond et al., 2020; Tort et al., 2020).

### 3.1 Requirements for Dressings

Natural polymeric materials that have been tested so far as wound dressings include agar, sodium alginate, hyaluronic acid, chitin, chitosan, carrageenan, cellulose, pectin, starch, and collagen. The variety of forms of dressings includes bandages, hydrogels, foil, sponges, foams, nanofiber mats (Zheng et al., 2020). Conventional dry dressings such as absorbent gauze and/or absorbent cotton dressings have a limited therapeutic effect and require multiple dressing changes, adversely affecting the patient’s experience (Stoica et al., 2020).

One of the main external factors responsible for optimal wound healing is hydration. Creating a moist wound environment through the use of ointments or hydrogels results in an improvement and acceleration of healing, a reduction in pain and a significant reduction in scarring compared to conventional wound treatment (Zhang et al., 2020). Moist wound healing optimally supports and facilitates the cleansing of the wound by the body, creating a clean wound environment. In the case of fibrin and necrotic tissues, they are softened and removed. Exudate, dead tissue debris and bacteria are absorbed through the dressing.

Besides additional active substances, a good modern dressing should support the wound healing process, which an ideal matrix/carrier in the dressing material should ensure (Bielecki et al., 2012; Zheng et al., 2020):

1. appropriate humidity—(favorable WHC to WRC ratio)
2. absorption of excess exudate (favorable WHC to WRC ratio)
3. maintaining an appropriate exchange of gaseous water vapor and gases (appropriate porosity)
4. not causing allergic reactions
5. not triggering the body’s immune response (inflammation in the initial stage of healing is desirable to stimulate the mechanisms of proliferation, i.e., rebuilding damaged tissues, but its prolongation may lead to the formation of a chronic wound)
6. biocompatibility
7. protecting the wound against bacterial infections (also as a physical barrier)
8. adequate thermal insulation (thickness of the dressing)
9. easy application and removal from the wound (adequate flexibility and mechanical strength)
10. painless dressing change (proper adhesion to the cells of regenerating tissues)
11. removal of necrotic cells (proper adherence to the wound, appropriate flexibility, size, adaptability to anatomical shapes)
12. observation of the healing process (desired transparency)

### 3.2 BC as a Dressing

Native bacterial cellulose can absorb wound exudate while providing good moisture of the wound bed and gas exchange with the environment. Since the 1990s, when this biomaterial was first used in medicine, much evidence has been accumulated to confirm that natural hydrogel created by bacterial cellulose supports wound healing (Czaja et al., 2004). A particular advantage of this material is that it is easy to obtain large sheets to cover the whole body and to adapt to the contours of the target area. In addition, due to its mechanical properties, this material also supports the skin tissue regeneration stage due to proper adhesion and support for cell proliferation and differentiation, and protection against mechanical injuries (Vilar et al., 2016; Emre Oz et al., 2021).

Native BC may constitute a physical barrier to secondary wound infections but has no bactericidal or bacteriostatic properties. To achieve this feature of the dressing, the BC membrane can be soaked with antibiotics (or other biocides) or subjected to further modification leading to the formation of a composite, e.g., with silver nanoparticles (Wu et al., 2014; Wen et al., 2015). The following section presents examples of the applications of BC as a dressing validated in *in vivo* experiments or clinical trials (Table 3).

**TABLE 3:** Characteristics of BC and BC composites used in wound care—results of clinical trials.

Material	Material characteristics	BC Manufacturer	Study design	Studied organism	Wound Type	Wound evaluation (changes assessed in wounds)	Clinical outcome	References
**Wound dressings**								
BC	tensile strength of 400.60 ± 51.19; elongation at break (%) of 9.56 ± 5.32	Gluconacetobacter xylinum (strain not provided)	*in vivo*	ICR male mice with an average body weight of 25–30 g	incised wound	• ability to heal wounds • histopathological examination	• faster and better wound healing (wound area treated with BC and BC-Vac was .56 and .5 mm, respectively, and controls were approximately 3 mm) • a wound covered with BC or BC-Vac showed better fluid retention compared to controls • easier removal of BC and BC-Vac dressing from the wound compared to control • more significant activity of fibroblasts and epithelialization	Qiu et al. (2016)
BC-vaccarin composite (BC-Vac)	tensile strength of 459.73 ± 48.21 and elongation at break (%) of 19.36 ± 10.45							
BC	ND	*Zoogloea* sp; POLISA, Biopolymers for Health, a startup hosted by the Federal Rural University of Pernambuco (UFRPE) (BC obtained from sugar cane)	clinical trial	24 patients aged around 42	preserve the nail bed after avulsion	• macroscopic wound observation • patient’s satisfaction • pain intensity	• increased patients’ satisfaction • reduced pain • faster wound healing process • protection against infections • good wound coverage • protection against mechanical injury	Oliveira et al. (2020)
BC	ND	ND	clinical trial	24 patients 49–90 years old	ischemic wounds after lower limb revascularization	• macroscopic wound observation	• faster wound healing process	Maia et al. (2019)
**Burn wound dressing**								
BC composite with silver sulfadiazine nanoparticles (BC—SSD membrane)	ND	BC membranes were purchased from Hainan Yida Food Co. Ltd (China).	*in vivo*	Wistar rats (weight ∼ 250 g)	partial-thickness skin wounds (20 × 20 mm)	• macroscopic examination of the wound surface • histopathological examination • microbiological examination of the wound surface	• no infection reduction in the number of bacteria on the wound surface • faster wound healing (wound healing 92.35% for BC-SSD and 78.83% for control) • sooner onset of re-epithelization compared to the control wound	Wen et al. (2015)
BC	the crystallinity of BC in AgNP-BC is about 83.68%	Hainan Yida Food Co. Ltd (China)	*in vivo*	Wistar rats (half male and half female) weight ∼250 g	deep partial-thickness 2nd-degree burn wound (80 °C) (20 mm–20 mm)	• macroscopic examination of the wound surface • histopathological examination	• less inflammation • faster wound healing • after 10 days, the wound defect filling was 62.13% (for AgNP-BC), 42.82% (for BC), 27.94% (for controls) (*p* < .05) • after 21 days, the wound defect filling was 99.3% (AgNP-BC), ∼ 82.37% (BC), 68.36% (control) (*p* < .05) • after 28 days, the wound defect filling was 100% (for AgNP-BC), about 96.42% (for BC), 81.58% (for control) • supporting the wound healing process • fewer bacteria on the wound surface (less bacterial multiplication) after 4 days 40.00 × 10^3^ CFU cm^−2^ (AgNP-BC), 128.13 × 10^3^ CFU cm^−2^ BC and 161.48 × 10^3^ CFU cm^-2^ (control) • extension of epidermal tissue deeper into the wound site (the thickness of the regenerated fresh epidermis and dermis was 111 and 855 μm, respectively (AgNP-BC, 74 and 619 μm (BC), 57 and 473 μm (control))	Wu et al. (2014)
AgNP-BC composite								
Composites of BC with Ag nanoparticles (BC-PDAg)	water vapour transmission rate arround 500 g/m^2^/day no influence of Ag ions on BC-PDAg composite permeability)	*Acetobacter xylinum* (NCIM 2526)	*in vivo*	female albino Wistar rats	third degree burn wounds	• macroscopic examination of the wound surface • histopathological examination • quantitative real time polymerase chain reaction (qRT-PCR)—expression level testing for the following genes: IL-10, VEGF-A, VEGF-B and bFGF, IL-1 α, IL-3, TGF-β3 and SMAD-3	• no allergic reactions • less inflammation • faster wound healing with no scar formation • facilitated colagenisation and granulation tissue formation as well as re-epithelization • on 20th day surface of healed wound was 94.35%, BC-PDAg 74.58% BC and 65.35% in control group • complete wound healing without scar formation in 25^th^ day (after BC-PDAg usage) • fast wound healing rate of BC-PDAg treatment through the upregulation of IL-10, VEGF-A, VEGF-B and bFGF and suppression of IL-1 α and IL-3 transcripts. Further, TGF-β3 and SMAD-3 expression has proven the promotion of wound healing without scar formation.	Jiji et al. (2020)
Composite of BC with polyhydroxyalkanoates (PHAs) BC/P(3HB/4HB), BC/P(3HB/4HB)/actovegin, P(3HB/4HB)/BC/fibroblasts	Young's modulus of BC (47.60 ± 6.32 MPa), BC/P(3HB/4HB) (65.08 ± 7.1 MPa), Water vapor transmission rate of BC, g/m^2^/d 2655 ± 21 and BC/P(3HB/4HB) (5014 ± 20) Fracture strength [MPa] of BC (.11 ± .13) and BC/P(3HB/4H) (.88 ± .08)	*Komagataeibacter xylinus* B-12068	*in vivo*	female Wistar rats	third-degree skin burns	• macroscopic examination of the wound surface • histopathological examination • biochemical and molecular methods of detecting factors of angiogenesis, • inflammation, type I collagen, and keratin 10 and 14	• faster and more effective wound healing • on 14th day wound surface reduced to 30,6% and 7.3% of initial wound surface after administration of BC/P(3HB/4HB)/Actovegin and BC/P(3HB/4HB)/fibroblasts • average healing rate for BC/P(3HB/4HB)/Actovegin and BC/P(3HB/4HB)/fibroblasts was estimated as .19 cm^2^/day and .4 cm^2^/day in control • more effective epidermization after treatment with BC/P(3HB/4HB)/Actovegin and BC/P(3HB/4HB)/fibroblasts	Volova et al. (2019)
cellulose-g-poly (acrylic acid) hydrogels	highly porous (80.3 up to 255 μm) hydrogel with high swelling ratio	ND	*in vivo*	female Sprague-Dawley rats	partial-thickness skin burns	• macroscopic examination of the wound surface • histopathological examination	• no signs of local inflammatory responses • faster wound healing process • improved epithelialization and faster fibroblast proliferation	Pandey et al. (2017)
**Chronic wound dressing**								
BC	ND	*Zooglea* sp.; POLISA, Biopolymers for Health, a startup hosted by the Federal Rural University of Pernambuco (UFRPE) (BC obtained from sugar cane)	clinical trial	39 patients	Chronic venous ulcers (CVU)	• macroscopic examination of the wound surface • dressing exchange frequency • patient satisfaction	• ∼ 3 times lower frequency of dressing changes • faster healing process • no macerization • epithelization of the edges of the wound • good wound adherence	Silva et al. (2021)
				25 patients		• macroscopic examination of the wound surface • patient satisfaction	• pain sensation reduction • maintaining humidity • absorption of excess exudate • protection against infections • protection against mechanical injury	Cavalcanti et al. (2017)
**Wound dressing**								
BC	ND	*Zoogloea* sp.; Laboratory of Biopolymers at the Experimental Station of Sugarcane, Federal Rural University of Pernambuco (BC obtained from sugar cane Molasses)	phase II clinical trial	141 patients (children, adolescents and adults)	postoperative wound of male urogenital organs	• effectiveness and safety (irritation of the skin in the area of the dressing - feeling of warmth, itching, swelling, pain and congestion) • patient satisfaction level • the time of the dressing remaining on the wound • healing time	• safe in wound healing (no complications such as ischemia, infections) • easy to use (put on and take off the wound) • long time of use without the need to replace • removes exudate • creates a moist environment • protects against foreign substances • supports tissue regeneration	Vilar et al. (2016)

### 3.3 Examples of BC Dressings in Clinical Applications and *In Vivo* Experiments

**FIGURE 5:**
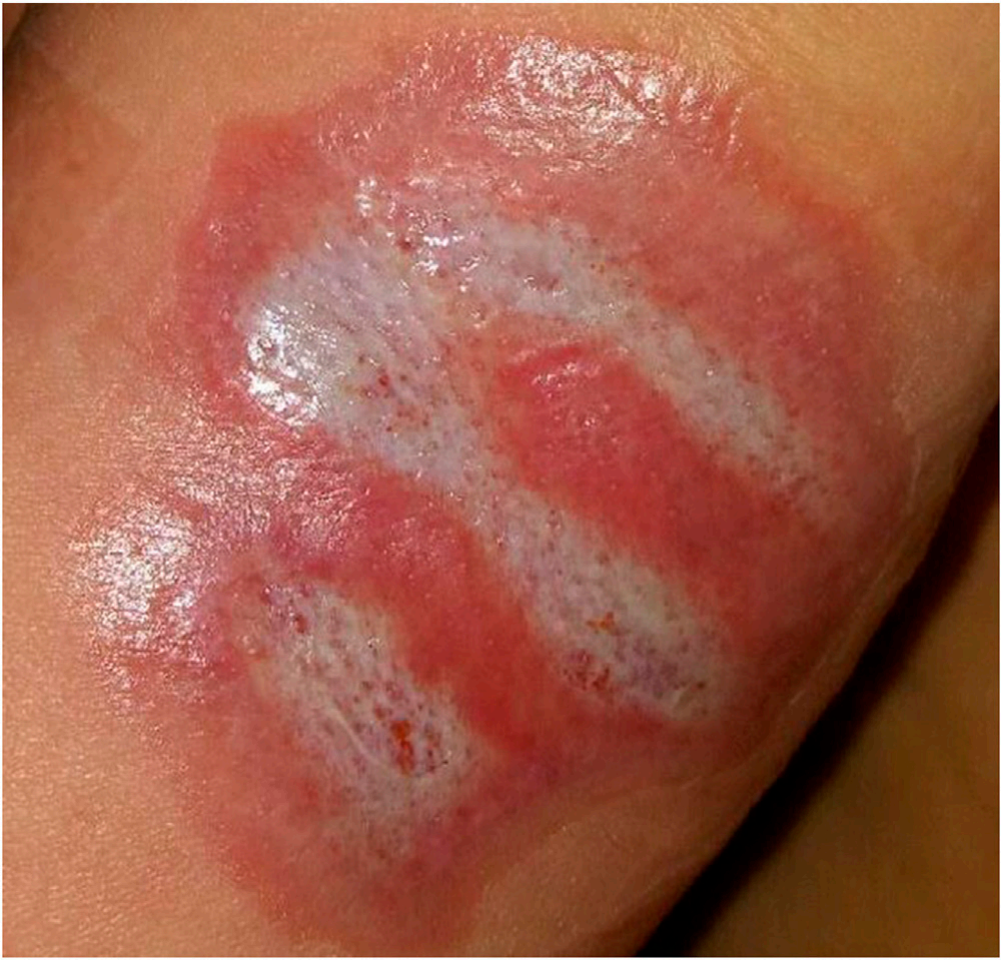
Wound 2 days after burn injury - prior to application of BC dressing.

**FIGURE 6:**
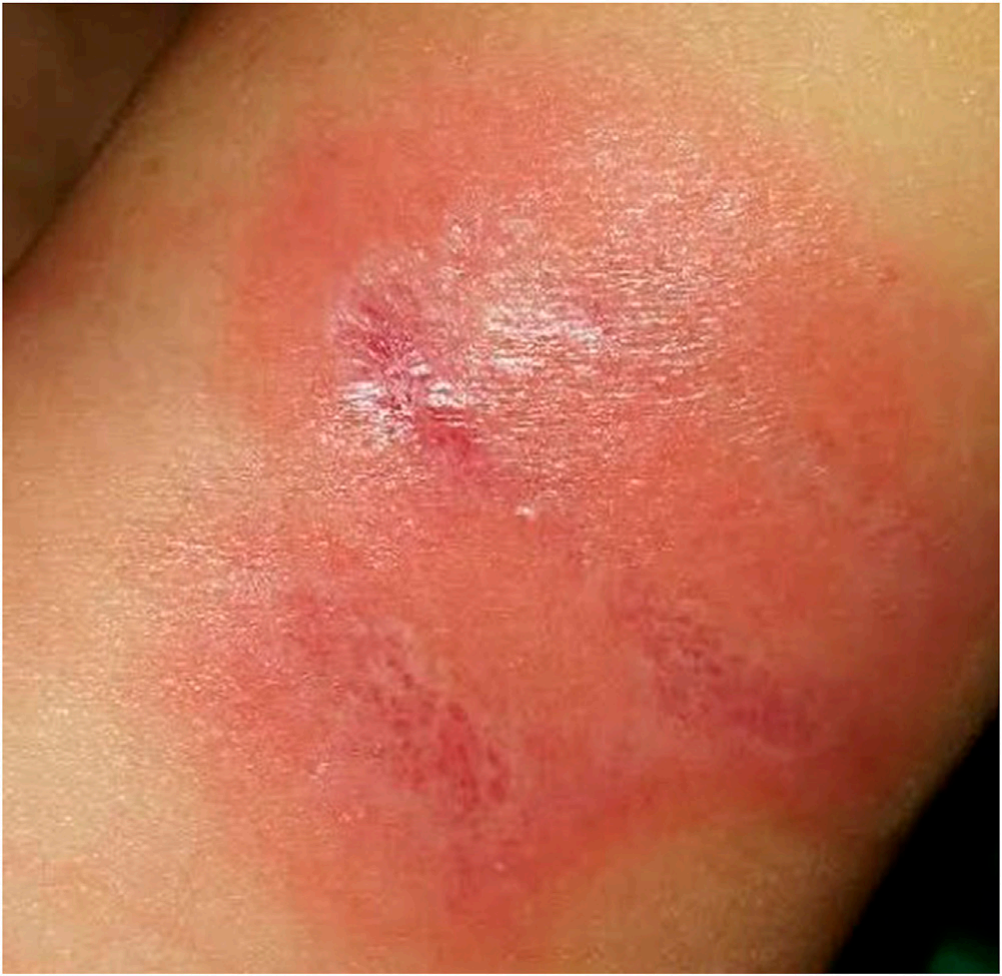
Wound after 3 weeks of using BC dressing.

Pioneering clinical trials of bacterial cellulose dressing were conducted at the Burn Wound Treatment Center in Siemianowice Śląskie, Poland (Czaja et all. 2020). They aimed to assess the suitability of the dressing in the treatment of burn wounds in the following areas: prevention of water loss through a burn wound, reduction of burn wound infection, preparing the wound for a skin graft. The study group consisted of patients with I, IIA, IIB and III degree skin burns covering 9%–18% of the total body surface. There were 77 study participants aged 18–70. The study results indicate that BC dressings are particularly effective for exuding wounds and wounds with elevated temperature. Due to the high water content, they cool the burn site and relieve pain. In the case of fresh and shallow burn wounds, BC dressing accelerates the epithelialization process, reducing the risk of infection and fluid loss. However, in the case of deep burns (IIB and III degree), it supports the removal of necrotic tissues from the wound. Faster healing was manifested by earlier change in gram-negative to gram-positive species abundance ratio as well. Thanks to proteolytic enzymes, gram-negative microflora causes faster wound cleansing, preparing for further treatment with a free skin graft (Czaja et al., 2004). According to clinical data, due to their flexibility, bacterial cellulose dressings are particularly recommended for the treatment of burn wounds in anatomically complex places (ears, face, joint area, hands, feet, genitals) (Oliveira et al., 2020). It has also been shown that combining the dressing with antiseptics, such as silver nitrate, hydroxyquinoline sulphate or boric acid, has a positive effect on reducing burn wound infection (Czaja et al., 2004). Clinical trials were also conducted by Jankau et al. using BC (Bowil Biotech Ltd., Władysławowo, Poland) as a dressing in a patient with thermal burns, achieving a very good clinical result (Figures 5, 6).

An example of a new application of bacterial cellulose dressings is their use in treating wounds in the urogenital area in men in the postoperative period. Phase 2 clinical trials showed that no adverse effects were observed in the case of applying a BC dressing, no disadvantages such as ischemia, infection and pain were identified, confirming the safety of the BC dressing and its effectiveness as a highly satisfactory alternative to the currently used dressings (Vilar et al., 2016).

A well-documented example of a BC composite studied *in vivo* in the process of wound healing is BC supplemented with vaccarin (BC-Vac), which performance has been compared to standard dressings, i.e., petrolatum gauze and a dressing with silver nanoparticles (Table 3). The most important advantages of the composite included increased flexibility, which facilitates its practical use (changing the dressing, adherence to the wound), and most importantly, a more advanced healing process (more active fibroblasts) was confirmed after 14 days of treatment (Qiu et al., 2016).

Rayon dressings are often used to treat chronic leg ulcers. When compared to rayon dressings for wound care, BC dressings have been shown (Silva et al., 2021) to be superior due to lower dressing change frequency, minimized direct contact with wounds, less risk of contamination and infection, greater patient autonomy and less dressing adhesion to bed (Silva et al., 2021).

Relieving inflammation and neuropathy in diabetic foot ulcers has been a great challenge for physicians for many years. Significant acceleration of the healing of diabetic foot ulcer wounds was achieved while using sheets of bacterial cellulose nanofibers soaked with venlafaxine (VEN) and doxycycline (DOX) as compared to the control group (Meamar et al., 2021). The patients showed no differences in their biochemical parameters before and after the intervention.

## 4 Prospects and Challenges for BC as a “Tailor-Made” Biomaterial for the Needs of Medicine

The native properties of BC modulated as in the discussed above *in situ* and *ex situ* modifications make it an attractive material for modern dressings and implants. However, constant progress in reconstructive medicine requires the development of more advanced, dedicated solutions. This section shows the emerging possibilities and pathways for further dedicated modifications of BC properties.

### 4.1 Molecular Intervention in BC Properties

Progress in functional genomics and transcriptomics of bacteria of the genus Komagataeibacter (Ryngajłło et al., 2019a, 2019b, 2020; Jang et al., 2019; La China et al., 2020) suggests that in the near future, the understanding of the relationship between the genotype of the producer strain and the properties of the polymer produced by its cells will reach a level that allows for the planned editing of the genome depending on the requirements set for materials in a specific application. The first published results of the properties of BC membranes produced by producer strain mutants confirm this thesis.

A correlation was observed between the thickness of the fibers and the mechanical properties of the membranes of the *K. obediens* R 37-4 and R37-9 mutants—the thinner the fibers, the higher the tensile strength (Taweecheep et al., 2019). BC synthesized by these mutants showed two times higher density and tensile strength than BC synthesized by the parent strain. The two obtained mutants, R 37-4 and R37-9, were also more efficient at synthesizing cellulose membranes (Table 3).

The data collected by our team indicate a correlation between changes in cell morphology and the diameter of cellulose fibers in the final material (Jacek et al., 2019a; 2019b, 2021). In the studies of the membrane structure using the SEM technique, thinner fibers (on average 45 nm in diameter in the lower part of the membrane) were observed in the case of shorter cells, such as those of the *K. xylinus* E25 strain (approx. 3.0 µm) (Jacek et al., 2021) or *K. hansenii* 23769 (approx. 2 µm) (Jacek et al., 2018) and widened (an average of 90 nm diameter in the lower part of the membrane) for longer cells in strains such as *K. hansenii* ATCC 5358 (5.2 µm) (Jacek et al., 2019a). In addition, studies have shown that by influencing the length of cells and their motility, it is possible to obtain a material with changed fiber dimensions and fiber arrangement in mutants of all the strains mentioned above within the *motA* and *motB* genes (Jacek et al., 2018; 2019a). The *motA* and *motB* genes have no proven molecular function in *Komagataeibacter* cells but are sequentially similar to the genes encoding the proton pumps necessary to generate the pmf (proton motive force) that drives flagella in *E. coli* (Hosking et al., 2006; Kim et al., 2008) and their homologues with proven function, e.g., in gliding in *Myxococcus* (Nan et al., 2013; Fu et al., 2018). Their presence is necessary to maintain motility in the strain *K. hansenii* ATCC 53582, as shown by microscopic observations and soft-agar motility assay for *motAB* deletion mutants (Jacek et al., 2019a). Moreover, these deletion mutants are characterized by a compact structure of thin fibers (average diameter of 40 nm compared to 90 nm on average for the wild type strain). As a consequence of the changes in the fiber architecture visible in the SEM examination, a change in mechanical properties was also noted, especially clearly in the case of Young’s Modulus for compressive tests of the wet membranes: it was ∼333 MPa for the disruptive strain, compared to ∼194 MPa for the wild type strain (Table 4) (Jacek et al., 2019a). Quite to the contrary, the motAB+ overexpression mutants obtained in the strain *K. hansenii* ATCC 23769 have elongated cells or occur in chains and produce cellulose material with a looser arrangement of fibers (Jacek et al., 2018). This kind of material was demonstrated to be useful in tissue engineering for the generation of scaffolds—the favorable difference in favor of BC derived from the mutant strain compared to the wild one was noticeable even at the level of the response of ATDC5 cells seeded into the scaffold (glycosaminoglycan secretion through Alcian blue staining) (Jacek et al., 2018).

**TABLE 4:** Characteristics of BC produced by *Komagataeibacter* mutants.

Bacterial strain	Genetic change/modification	Average BC yield (g dry weight L^−1^)	Density (g/cm^3^)	Average BC diamete*r* (nm)	Tensile strength (MPa)	Young’s modulus (GPa)	References
*K. obediens* MSKU 3 (parental strain)	—	.33	.43 ± .05	70.52	73.94 ± 16.94	5.83 ± .69	Taweecheep et al. (2019)
R-30-3	frameshift mutation in *bcsCI* encodes cellulose synthase subunit CI	2.15	.42 ± .02	65.9	43.56 ± 10.98	3.60 ± .40	
R-30-12		.53	.54 ± .01	70.14	73.42 ± 5.56	5.14 ± .58	
R-37-4		1.12	.72 ± .03	59.14	158.72 ± 28.29	8.75 ± 1.54	
R-37-9		.7	.85 ± .07	34.58	159.47 ± 29.76	9.83 ± .69	
*K. hansenii ATCC* 53582 (parental strain)		6.28 ± .009		91 ± 23		6.86 ± 1.6 × 10^–3^	Jacek et al. (2019a)
A:kan	Disrupted *motA*	1.73 ± .004		45 ± 12		5.36 ± .9 × 10^–3^	
AB:kan	Disrupted *motA* i *motB*	1.36 ± .002		41 ± 12		5.03 ± 1.3 × 10^–3^	
*K. hansenii* ATCC 23769 (parental strain)			1.25 ± .12	50–70	.487 ± .116	2.41 ± 5.9 × 10^–3^	Jacek et al. (2018)
motAB+	Overexpression *motA* and *motB*		1.03 ± .09	80–110	.532 ± .110	3.02 ± 9.8 × 10^–3^	

### 4.2 BC as a Drug Delivery System—Potential and Challenges

Among the many listed medical applications of bacterial cellulose, its use in developing drug delivery systems (DDS) is the most diverse application field. Considering the different types of drug administration to the patient’s body (including dermal, transdermal and oral administration) and the different types of conditions managed with such treatments, a large number of published studies focus on an attempt to create an effective BC-based system, enabling the effective delivery of selected drugs to a wound or diseased tissue (Ciolacu et al., 2020). Such systems involve carriers based on whole fragments of membranes and its composites, cellulose beads formed in shake cultures, as well as cellulose fibers, which are used for creating hybrids for drug immobilization (Shi et al., 2014; Shao et al., 2016; Bayazidi et al., 2018; Cacicedo et al., 2018; Blanco et al., 2021). The latest trends in this area are oriented towards developing controlled systems targeting specific areas undergoing treatment and stimulating specific factors to reduce the adverse effects of the drug on healthy tissues. It is also expected that the carriers and their degradation products will be harmless to the body, able to accommodate the amount of the drug necessary to achieve the expected treatment outcomes and that the release time of such drugs will be adjustable to the duration of treatment, be it hours or even days (Ciolacu et al., 2020). In this field, bacterial cellulose has considerable potential as it is a highly hydrophilic biopolymer, which is relatively easy to modify and create various types of composites and hybrids, and above all, shows high biocompatibility. Nevertheless, published studies report on various challenges that still face scientists who explore BC-based DDS. First of all, cellulose sheets are not easy to handle in forming standard and well-known drug carriers, such as liposomes, conjugates or polyplexes, for instance. In most published studies, a membrane is used as a carrier and most often as a dressing, so the drug is delivered by soaking, which limits the possibility of controlled, delayed release (Silva et al., 2014; Cacicedo et al., 2020). Due to the compact nanostructure resulting from the dense cross-linking of the fibers, the incorporation of larger particles into the membrane is also challenging. The vast majority of research into BC carriers concerns combining cellulose (most often a dressing) with small molecules, such as analgesics and anti-inflammatory agents (e.g., diclofenac, ibuprofen, venlafaxine and doxycycline), bacteriostats such as metal ions (TiO_2_, ZnO_2_, Ag, etc.), antibiotics (amoxicillin, ciprofloxacin, gentamicin, etc.), or other chemical compounds (e.g., glycerol, glutaraldehyde, ε-poly-l-Lysine, nisin, ocetnidine, rutin) (Silva et al., 2014; Shao et al., 2016; Anton- Sales et al., 2019; Gorgieva and Trček, 2019; Jiji et al., 2020; Blanco et al., 2021; Emre Oz et al., 2021; Fonseca et al., 2021; Meamar et al., 2021). Larger molecules such as proteins (e.g., albumin, lysozyme, lipase or phospholipase) and growth factors should be adequately immobilized in the membrane after modifying the porosity of the cellulose nanostructure (Wu et al., 2017; Drozd et al., 2019; Wang et al., 2020). For such immobilization, carriers made of bacterial cellulose nanofibers (BCNF) are often used when porosity can be adjusted at the stage of carrier development or when the immobilization takes place during its development (Bayazidi et al., 2018). Such techniques find particular application in the development of cellulose dressings and implants, fostering angiogenesis and epithelialization. Promising solutions include cellulose carriers that stimulate blood vessel formation with the help of, e.g., vascular endothelial growth factor (VEGF) or interleukin 4 (IL-4), as well as dressings and implants supporting healing by inhibiting pro-inflammatory factors such as matrix metalloproteinases (Ai et al., 2020; Li et al., 2020; Wang et al., 2020). Stopping the expression of genes coding selected MMPs with the use of siRNA molecules encapsulated in bacterial cellulose and its hybrids, will allow for avoiding long-term treatment or hard-to-heal wounds and reducing inflammation in the case of surgical interventions. Moreover, this type of gene therapy will reduce the adverse effects of drugs on other tissues.

The most advanced, although still relatively few targeted therapies that use bacterial cellulose are applicable to cancer treatment, where the active substance is usually doxorubicin, (Cacicedo et al., 2018; Ando et al., 2021; Islam et al., 2021). They use cellulose nanofibers (BCNF) or hybrids of BC hydrogel with nanostructured lipid carriers for immobilization to efficiently and effectively release the drug at the target area. In this field, the challenge is in functionalizing cellulose in such a way as to make it an actual release system. Hence, new, non-aggressive methods of increasing cellulose reactivity (e.g., enzymatic oxidation) or the development of composites, e.g., with magnetic, thermo- and photosensitive properties, (Ivanova et al., 2020; Kim et al., 2020; Troncoso and Torres, 2020; Poddar and Dikshit, 2021; Sriplai and Pinitsoontorn, 2021). This direction of research may lead to developing a carrier that will be non-toxic, functional and adapted to the state-of-the-art treatment methods.

## 5 Conclusion

The Human body in large is composed of soft tissues. They play an important role in maintaining the human body with structure and function. Repairing or replacing them is now an important part of reconstructive surgery. For this purpose, many organic or inorganic polymers are used from which the structures that make up the human body are built or rebuilt. One of these polymers is biocellulose, produced by *Komagataeibacter* batteries.

Searching the bibliographic databases: Scholar, Embase or Europe PMC from the last 5 years using the tool “LinkSource” and typing the keywords: bacterial cellulose, biocellulose, bionanocellulose, BNC or nanocellulose in combination with the keyword reconstructive surgery, we get over 62, 452 results. In the PubMed online database, typing in the same keywords, only 2,843 papers appear, and narrowing the search to humans, only 27 papers appear. Even if we assume that some of them are duplicated, it shows the importance of the problem. Thus, the potential of using bacterial cellulose in soft tissue reconstruction, from reconstruction to aesthetics, is unlimited. An interesting and often misunderstood term in the context of cellulose is the acronym BNC, in medicine and surgery it stands for bladder neck contracture. We excluded this from our search. Scientific papers describing clinical use of CC in animals or humans, however, are sparde, indicating how much this topic is not researched.

Possibilities of using BC in modern and reconstructive surgery are enormous. As Table 2 shows, many surgical disciplines have the chances to benefit from the potential of this natural polymer. It is a desirable, versatile material thanks to its various shape, hydratation, flexibility, hardness . Its use requires research, especially phase III studies. Unfortunately, even the models of pigs are not a proper equivalent of the metabolism of the human body, but even these are few. This is particularly when we mention about implanted parts into animal or human body.

The purpose of this review is to highlight the challenge for surgeons and biotechnologists to produce a cellulosic material that will meet the versatile needs of soft tissue restoration as well as use as inserts in various forms (e.g., skeletons, frames or structures, tubes, and even valves) in the human body. Such material is yet to be developed, as shown in the above article, bacterial cellulose has a chance to come close to the ideal. In the above review, the authors combined the knowledge of surgeons and biotechnologists to present the expectations and possibilities of microbial cellulose, believing it will be possible to personalize it and combine it with other substances to induce the growth of regenerated tissues and be “indestructible.” Therefore, further research activities need to be conducted to fill current gaps from laboratory *in vitro* and *in vivo* research to tests in humans and put in commercial production, so that unlimited cellulose inserts will replace missing soft tissues, both in their form and function. Above all authors believe that patient directed “bacterial cellulose” will certainly improve their quality of life. The authors also hope that further research and production capabilities will allow for quick and inexpensive production, including 3D printing, of unlimited cellulose inserts to replace soft tissues, both in their function and form. This, however, requires close cooperation between the interested parties and the patients.
